# Co-expression of SARS-CoV-2 entry genes in the superficial adult human conjunctival, limbal and corneal epithelium suggests an additional route of entry via the ocular surface

**DOI:** 10.1016/j.jtos.2020.05.013

**Published:** 2021-01

**Authors:** Joseph Collin, Rachel Queen, Darin Zerti, Birthe Dorgau, Maria Georgiou, Ivo Djidrovski, Rafiqul Hussain, Jonathan M. Coxhead, Agatha Joseph, Paul Rooney, Steven Lisgo, Francisco Figueiredo, Lyle Armstrong, Majlinda Lako

**Affiliations:** a Biosciences Institute, Faculty of Medical Sciences, Newcastle University, UK; b Microscopy Centre and Department of Applied Clinical Sciences and Biotechnology, University of L'Aquila, Italy; c NHS Blood and Transplant Tissue and Eye Services, Liverpool, UK; d Department of Ophthalmology, Royal Victoria Infirmary and Newcastle University, Newcastle, UK

**Keywords:** Cornea, Conjunctiva, sars-cov-2, ace2, tmprss2

## Abstract

**Purpose:**

The high infection rate of SARS-CoV-2 necessitates the need for multiple studies identifying the molecular mechanisms that facilitate the viral entry and propagation. Currently the potential extra-respiratory transmission routes of SARS-CoV-2 remain unclear.

**Methods:**

Using single-cell RNA Seq and ATAC-Seq datasets and immunohistochemical analysis, we investigated SARS-CoV-2 tropism in the embryonic, fetal and adult human ocular surface.

**Results:**

The co-expression of *ACE2* receptor and entry protease *TMPRSS2* was detected in the human adult conjunctival, limbal and corneal epithelium, but not in the embryonic and fetal ocular surface up to 21 post conception weeks. These expression patterns were corroborated by the single cell ATAC-Seq data, which revealed a permissive chromatin in *ACE2* and *TMPRSS2* loci in the adult conjunctival, limbal and corneal epithelium. Co-expression of ACE2 and TMPRSS2 was strongly detected in the superficial limbal, corneal and conjunctival epithelium, implicating these as target entry cells for SARS-CoV-2 in the ocular surface. Strikingly, we also identified the key pro-inflammatory signals TNF, NFKβ and IFNG as upstream regulators of the transcriptional profile of ACE2^+^TMPRSS2^+^ cells in the superficial conjunctival epithelium, suggesting that SARS-CoV-2 may utilise inflammatory driven upregulation of *ACE2* and *TMPRSS2* expression to enhance infection in ocular surface.

**Conclusions:**

Together our data indicate that the human ocular surface epithelium provides an additional entry portal for SARS-CoV-2, which may exploit inflammatory driven upregulation of *ACE2* and *TMPRSS2* entry factors to enhance infection.

## Introduction

In December 2019, several cases of pneumonia of unknown aetiology were identified in Wuhan, Hubei province, China [1]. On January 7th, the pathogen was identified as a novel enveloped RNA betacoronavirus2 and named severe acute respiratory syndrome coronavirus 2 (SARS-CoV-2) [2]. Since then, the outbreak has quickly expanded into an epidemic and the World Health Organization (WHO) declared coronavirus disease 2019 (COVID-19) a public health emergency on the 30^th^ January 2020 and subsequently a pandemic on 11^th^ March 2020. As of 22nd of April, 2,539,129 confirmed cases and 179,725 deaths have been recorded [3]. The virus, which spreads via human-to-human transmission is highly infectious and transmitted mainly through respiratory droplets and direct contact with infected persons, although the possibility of feco‐oral and other routes of transmission remains to be further assessed. At present, the average mortality rate amongst the affected individuals is 6.9% and the number of infected people is on the rise [3]; hence, COVID-19 poses a huge threat to the global population.

SARS-CoV-2 is a single-strand RNA virus, with a genome around 30 kb in length, with 79% similarity to SARS-CoV at the nucleotide level [4]. The genomes of all known Coronaviruses (CoVs) code for 4 main structural proteins: (1) spike (S) protein, which is responsible for attachment to host receptors; (2) membrane (M) protein, which helps to shape the virion particles and binding to nucleocapsid; (3) envelope (E) protein, which plays a role in the assembly and release of particles and (4) nucleocapsid (N) protein, which facilitates the binding of the genome to a replication-transcription complex that is required for the replication of genomic material [5].

Entry of CoVs into the target cells is dependent on the binding of S protein to a cellular receptor and subsequent priming of S protein by host cell proteases. Similarly to SARS-CoV, SARS-CoV-2 uses angiotensin converting enzyme 2 (ACE2) as receptor for entry into the cells [[6], [7], [8], [9]]. Binding of the surface unit S1 of S protein facilitates viral attachment to the surface of target cells. The transmembrane protease serine type 2 (TMPRSS2) cleaves the S protein at the S1/S2 and the S2’ site and allows fusion of viral and cellular membranes, enabling entry of CoV into the host cell [8]. In the absence of proteases at the cell surface, CoVs can enter the cells via the endosomal pathway and S protein is activated for fusion by cathepsin B/L in the endosome, although viral replication is 100 times less efficient than direct viral cell entry mediated by proteases [10]. Recent work has shown that ACE2 is expressed in multiple epithelial cell types across the airway [11], with highest expression in the nasal goblet/secretory cells and ciliated cells, consistent with the main pathology of COVID-19.

Currently the potential extra-respiratory transmission routes of SARS-CoV-2 remain unclear, although there is increasing clinical and scientific evidence that human ocular surface may represent an additional route of entry. In a recent study of 38 patients clinically confirmed with COVID-19, 31.6% had ocular manifestations consistent with conjunctivitis and 16.7% of these had positive results for SARS-CoV-2 from both conjunctival and nasopharyngeal swabs [11]. A recent case report from China described a clinician who became infected with SARS-CoV-2 whilst wearing an N95 mask but no eye protection, which suggests that the ocular surface could also serve as an additional entry route for the virus [12]. Other case reports corroborate these findings as many ophthalmologists around the world have contracted COVID-19 through routine eye examination of infected patients, who are often asymptomatic [12]. Indeed, some respiratory viruses, including SARS-CoV, have been shown to cause ocular implications (conjunctivitis) in infected individuals and to establish a respiratory infection following ocular exposure [13]. A recent publication suggests that Rhesus macaques can be effectively infected with SARS-CoV-2 via the ocular surface conjunctival route [14]. Numerous properties allow the eye to serve as a potential site of virus replication as well as a gateway for the establishment of respiratory infection. This is primarily enabled by the nasolacrimal system, which provides an anatomical bridge between ocular and respiratory tissues [15], facilitating the drainage process of contaminated tear fluid containing the virus from the ocular surface, into the nose and then into respiratory tract tissue. And, similar to human respiratory tract epithelium, human ocular surface conjunctival epithelium secreted mucins contain sialic acids, which bind the S protein, facilitating CoVs infection [16].

A number of published studies show that *TMPRSS2* is expressed in the epithelium of a large number of tissues including the prostate, upper airways and lung, the kidney, pancreas, colon, salivary gland, stomach, small intestine, bile duct, ovaries among other tissues [17]. High expression of the *ACE2* receptor is also detected in the oral mucosa [18] and nasal epithelium, lung alveolar epithelial type II cells, liver cholangiocytes, colon, esophagus, ileum, rectum, stomach epithelial cells, and kidney proximal tubules [19]. Some of the components of the renin angiotensin system (RAS) including *RENIN, PRORENIN, ACE1* and *ANG II* have been investigated in the ocular surface and found to be expressed in the human conjunctiva and cornea [20], and some others such as ACE2 have been found in the aqueous humour. However, a detailed analysis of TMPRSS2 and ACE2 in the ocular surface during human development and adulthood is lacking, nonetheless this analysis is critically important for substantiating the hypothesis of SARS-CoV-2 infection via the ocular surface.

## Results and discussion

### Co-expression of SARS-CoV-2 entry factors in the adult human ocular surface epithelium and presence of inflammatory programs in the putative target cells

**Fig. 1:**
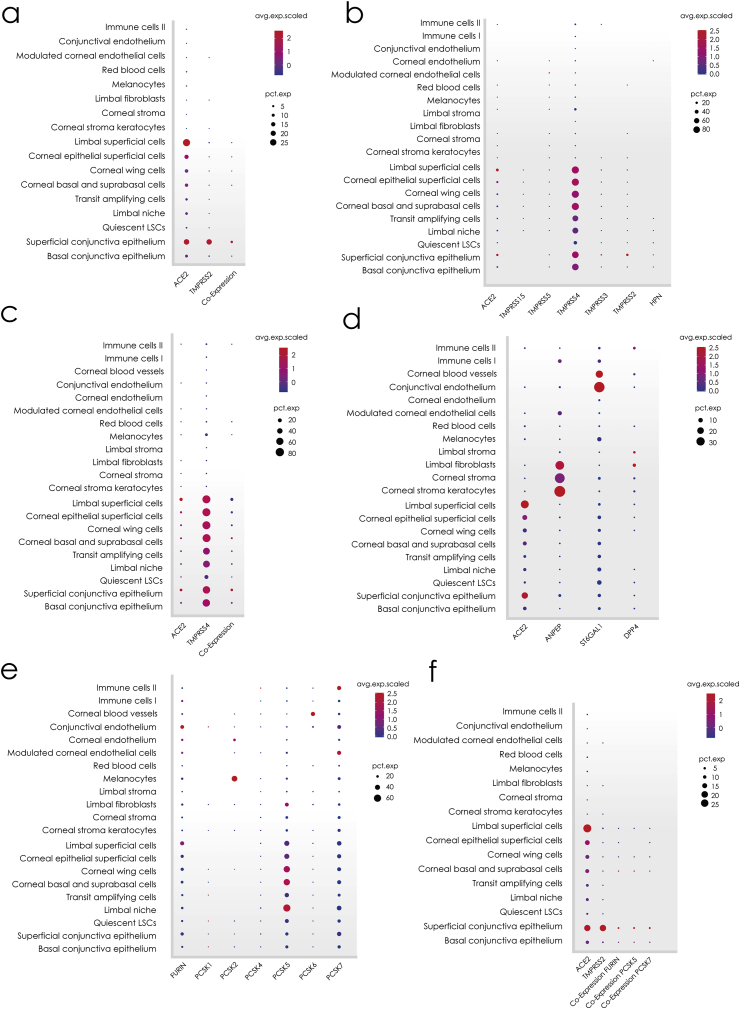
**Expression of *ACE2* and *TMPRSS2* in the adult human ocular surface and co-expression in the conjunctival epithelium**. **a)** RNA expression of SARS-CoV-2 receptor *ACE2* (first column), entry protease *TMPRSS2* (second column) and their co-expression (third column) in the human adult cornea and conjunctiva; **b)** RNA expression of *ACE2, TMPRSS2* and related family members in specific cell subtypes found in the human adult cornea and conjunctiva; **c)** RNA expression of SARS-CoV-2 receptor *ACE2* (first column), protease *TMPRSS4* (second column) and their co-expression (third column); **d)** RNA expression of *ACE2*, *ANPEP*, *ST6GAL1* and *DPP4* in the human adult cornea and conjunctiva; **e)** RNA expression of *PCSK3 (Furin), PCSK1, PCSK2, PCSK4, PCSK5, PCSK6* and PCSK7; **f)** Co-expression of *PCSK3, PCSK5* and *PCSK7* with *ACE2* and *TMPRSS2*. a-f) Raw expression values were normalised, log transformed and summarised. The size of the dots indicates the proportion of cells, while the colour indicates the mean expression.

**Fig. 2:**
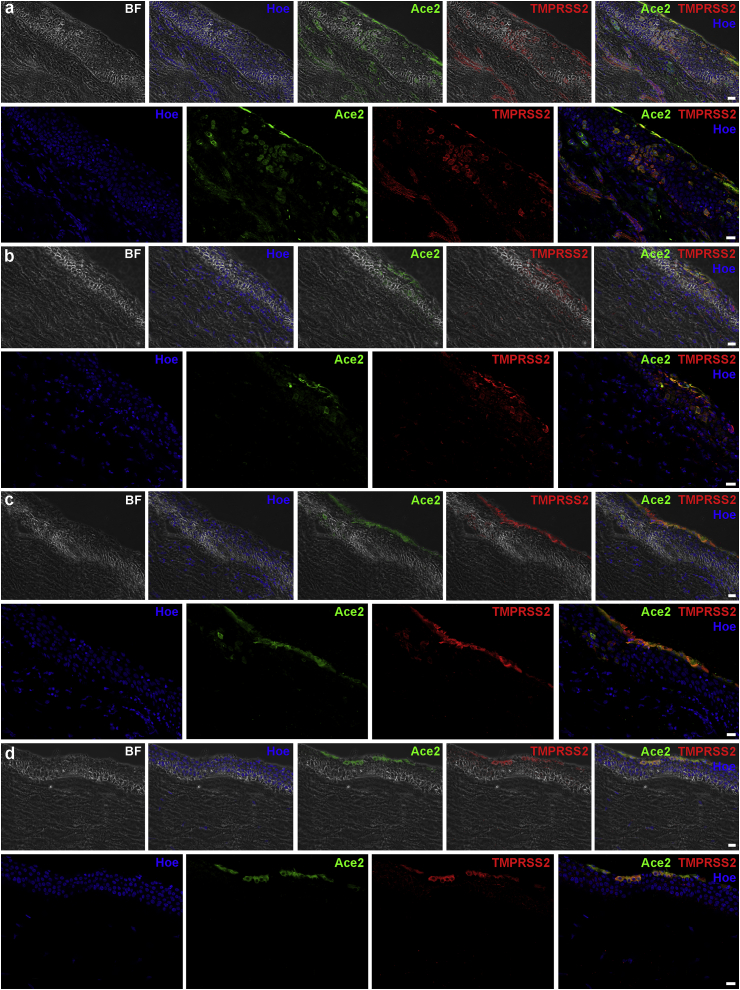
**Expression of ACE2 and TMPRSS2 in the ocular surface**. ACE2 and TMPRSS2 expression in the limbal **(a)**, peripheral cornea **(b)**, conjunctival **(c)** and central corneal epithelium. Hoe – Hoechst and BF – bright field. Scale bars 20um.

Our Human Cell Atlas analyses have shown that *ACE2* is expressed in multiple epithelial cell types across the airway, with highest co-expression with *TMPRSS2* in the nasal secretory cells, consistent with the main pathology of COVID-19 [21]. The co-expression of *ACE2* and *TMPRSS2* was also detected in the superficial conjunctival epithelial cells, which led us to assess in detail the expression of these genes and other proteases that may be involved in SARS-CoV-2 cellular entry via the ocular surface. Using our scRNA-Seq dataset of human adult cornea and conjunctiva, we found that *ACE2* was expressed in a large percentage of cells (8.7%) with highest expression in the limbal and conjunctival superficial epithelium (Fig. 1a). ACE2 protein expression was most highly detected throughout the limbal and peripheral corneal epithelium (Fig. 2a and b), in the superficial conjunctival epithelium as well as some basal and suprabasal cells (Fig. 2c) and in the superficial central corneal epithelium (Fig. 2d).

**Extended data Fig. 1:**
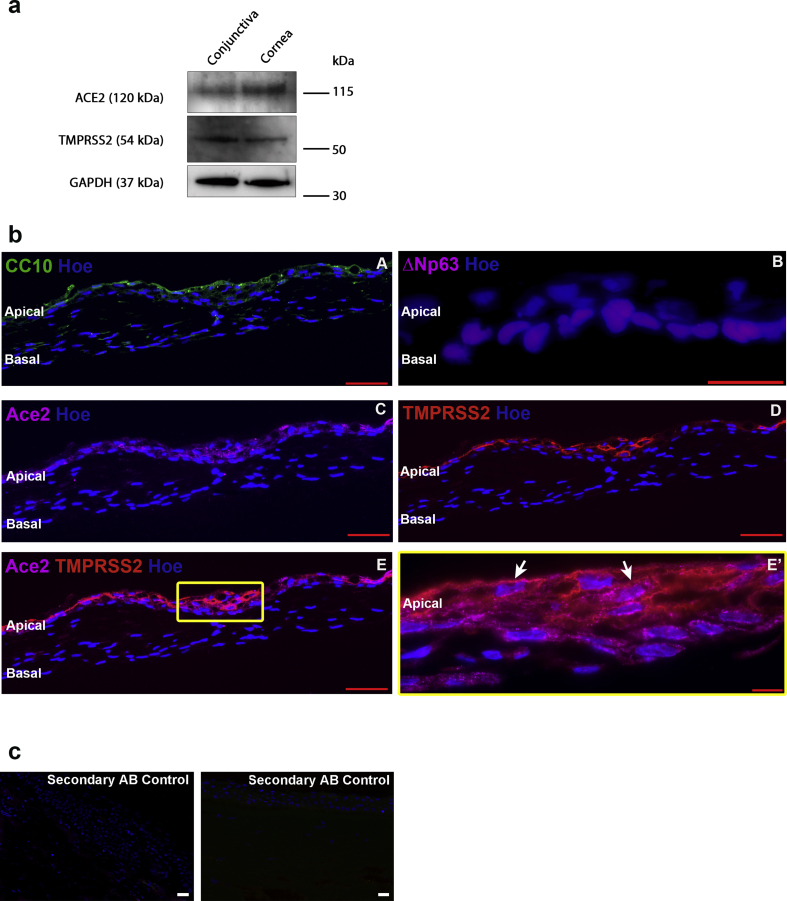
**ACE2 and TMPRSS2 expression in the ocular and *in vitro* lung derived tissue**. **a)** Western Blot analysis of ACE2 and TMPRSS2 expression in the corneal and conjunctival tissues. GAPDH is used as a loading control; **b)** Expression of ACE2 and TMPRSS2 and their co-expression in the apical surface (defined by the CCL10 expression) of human induced pluripotent stem cell derived lung organoids. The basal side is defined by deltaNp63 expression. The white arrows indicate ACE2+TMPRSS2+ cells. Scale bars: A-E 50 μm and E′ is 10 μm; **c)** Immunohistochemical analysis of limbal (left) and central cornea (right) panel with secondary antibodies; scale bars 20 μm. Hoechst staining is shown in blue colour.

*TMPRSS2* was expressed in fewer cells (2.15%) and was detected predominantly in the epithelial cells with highest expression in the basal and superficial conjuctival epithelium (Fig. 1a). Immunohistochemical analysis indicated the presence of TMPRSS2 immunopositive cells throughout the limbal and peripheral corneal epithelium (Fig. 2a and b), the superficial conjunctival epithelium as well as some basal and suprabasal cells (Fig. 2c) and the superficial central corneal epithelium (Fig. 2d). The specificity of the antibodies was confirmed by Western Blot analysis (Extended data Fig. 1a), positive immunostaining on the apical side of *in vitro*-derived lung tissue (Extended data Fig. 1b) and by removing secondary antibodies in the immunohistochemical analysis of ocular surface (Extended data Fig. 1c).

Superficial conjunctival epithelial cells showed the highest co-expression of *ACE2* and *TMPRSS2* (6.6% of the cells), followed by basal conjunctival epithelial cells (0.49% of cells). Another TMPRSS family member, *TMPRSS4* was also expressed in basal and superficial corneal, limbal and conjunctival epithelium (Fig. 1b). *TMPRSS4* was co-expressed with *ACE2* mainly in the suprabasal and basal corneal epithelial (9.6% of the cells) and superficial conjunctival epithelial cells (17.6% of the cells); however, the highest co-expression was observed in the superficial conjunctival epithelium (Fig. 1c). Co-expression of ACE2 with TMPRSS2 was detected throughout the limbal and peripheral corneal epithelium, superficial conjunctiva and some basal and suprabasal cells as well as superficial corneal epithelium (Fig. 2a–d). Together these data suggest that the superficial ocular surface epithelium may provide an entry portal for the SARS-CoV-2 entry.

**Fig. 3:**
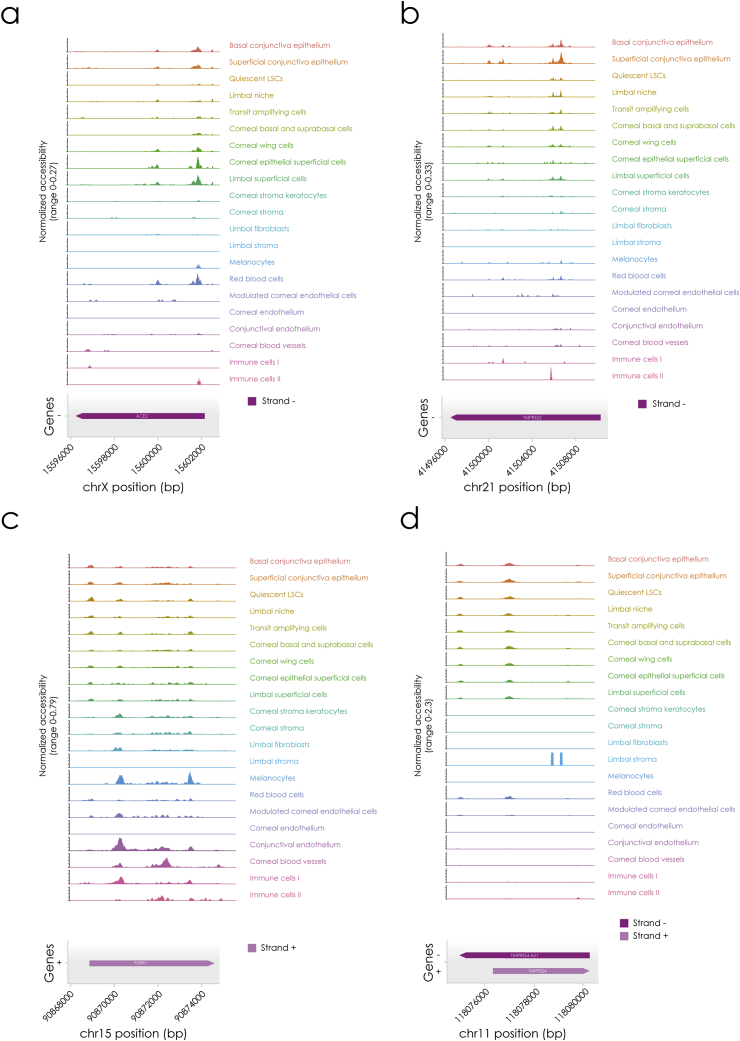
**Chromatin accessibility at the *ACE2, TMPRSS2, PCSK3 and TMPPRSS4* loci in the adult human ocular surface**. Schematic single cell chromatin accessibility of *ACE2***(a)**, *TMPRSS2***(b)**, *PCKS3***(c)** and *TMPRSS4* loci **(d)** in the human adult cornea and conjunctiva samples.

**Extended data Fig. 2:**
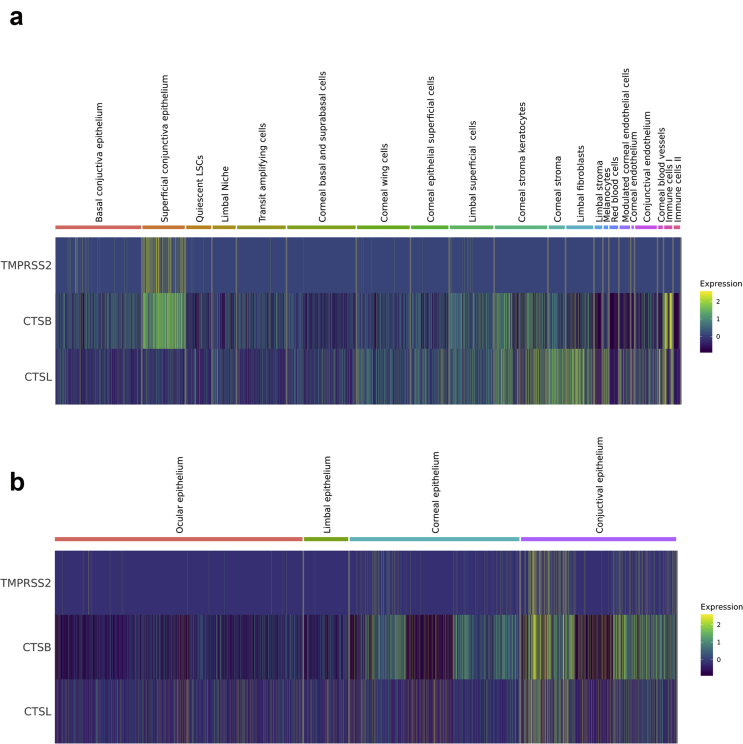
RNA expression of *TMPRSS2, CTSB* and *CTSL* shown as a gene expression heatmap for adult (a) and the embryonic and fetal cornea and conjunctiva cell type subsets (b).

We also assessed the expression of *ANPEP* (associated with HCoV-22944), *DPP4* (associated with MERS-CoV45) and the enzyme *ST6GAL1*, which is important for the synthesis of sialic acids recognised by the influenza viruses (Fig. 1d). The expression of all these three receptors was generally low in the corneal, limbal and conjunctival epithelium, compared to *ACE2*. Superficial conjunctival epithelium also expressed the highest level of *CTSB* (Extended data Fig. 2a), which has been suggested to facilitate viral transmission, albeit less efficiently than TMPRSS2^10^. A large number of proteases including proprotein convertases and trypsin have been shown to proteolytically process the spike protein in addition to TMPRSS2 and endosomal cathepsins [22]. Hence, we assessed the expression of *PCSK3* (Furin), *PCSK4, PCSK5 and PCSK7* as well as their co-expression with *ACE2* in our data set. *PCSK3*, *PCSK5* and *PCSK7* were the only proprotein convertases expressed in the limbal, corneal and conjunctival epithelium (Fig. 1e); these were also co-expressed with *ACE2* and *TMPRSS2* in a small subset of superficial and basal conjunctiva epithelium, superficial limbal and corneal epithelium (Fig. 1f) The *ACE2* expression is also supported by scATAC-Seq data, showing higher accessibility at the *ACE*2 locus in the superficial limbal and corneal epithelium as well as basal and superficial conjunctiva (Fig. 3a). The strong expression of *TMPRSS2* in the superficial conjunctival epithelium is also supported by the higher accessibility of *TMPRSS2* locus in these cells (Fig. 3b). This analysis also showed chromatin accessibility at the *PCSK3* and *TMPRSS4* loci on the basal and superficial conjunctival epithelium as well as quiescent limbal stem cells (LSCs), transient amplifying cells (TA) and corneal and limbal epithelium (Fig. 3c and d). No selective chromatin accessibility was observed for the other *TMPRSS* or *PCSK* family member's loci (*data not shown*).

**Extended data Fig. 3:**
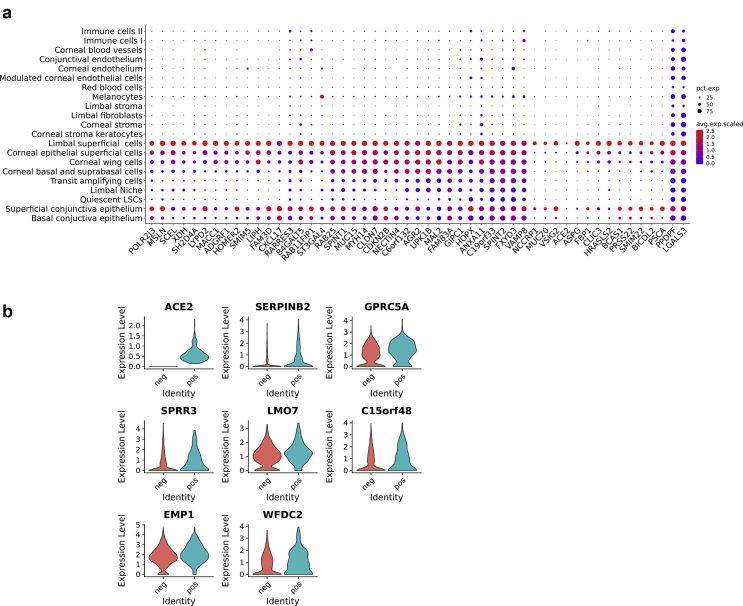
**Ocular surface genes associated with *ACE2* expression and transcriptional characterisation of ACE2**^**+**^**cells**. **a)** Ocular surface epithelium expression of the top 50 genes correlated with *ACE2* expression identified through Spearman's correlation analysis in the adult human corneal and conjunctiva dataset. The dot size represents the proportion of cells within the respective cell type expressing the gene and the colour represents the average gene expression level within the particular cell type; **b)** Violin expression profiles showing the expression of 10 most significantly upregulated genes in the ACE2^+^ expressing cells compared to ACE2^-^.

To identify genes associated with *ACE2* expression across all the cell types in the adult cornea and conjunctiva, we performed Spearman's correlation analysis and then selected the top 50 genes with the highest correlation coefficients. (Extended data Fig. 3a). This analysis revealed genes highly expressed in the superficial layer of the corneal, limbal and conjunctival epithelium. A secretory tissue specific signature was detected encompassing key markers of goblet cells (e.g. *MUC15, MUC2, AGR2, VAMP8* and *CLDN7*), alongside the mucosal chemokine *CXCL1*7 that attract immature dendritic cells and blood monocytes. Interestingly, a protease inhibitor that targets trypsin-like serine proteases involved in activation of both influenza viruses and metapneumoviruses, *SPINT2* was also detected amongst the top 50 *ACE2* correlated genes [23]. To gain more insights into the ACE2 expressing cells, a differential gene expression analysis between ACE2^+^ and ACE2^-^ cells was performed (Extended data Fig. 3b), revealing in the top 10 differentially expressed list genes involved in innate (*WFDC2* [24]*, LGALS3 and LMO7*) and adaptive immunity (*SERPIN*B2 [25]). CellphoneDB [26] analysis highlighted one significant interaction for ACE2 with the ghrelin and obestatin prepropeptide (GHLR), which encodes the ghrelin-obestatin preproprotein that is cleaved to yield two peptides, ghrelin and obestatin. This observation corroborates a recent report, which predicts direct interactions between ACE2 and GHLR [27]. Grehlin is an apetitite stimulant, thought to regulate multiple activities including hunger and glucose-secreted stimulation. In our dataset, *GHLR* expression was highest in the immune cells and no expression was detected in the conjunctival epithelium (data not shown). These interactions need to be validated experimentally to assess how higher levels of GHLR may influence ACE2 expression; nonetheless they resonate with the higher susceptibility of obese individuals to SARS-CoV-2 infection [28].

**Extended data Fig. 4:**
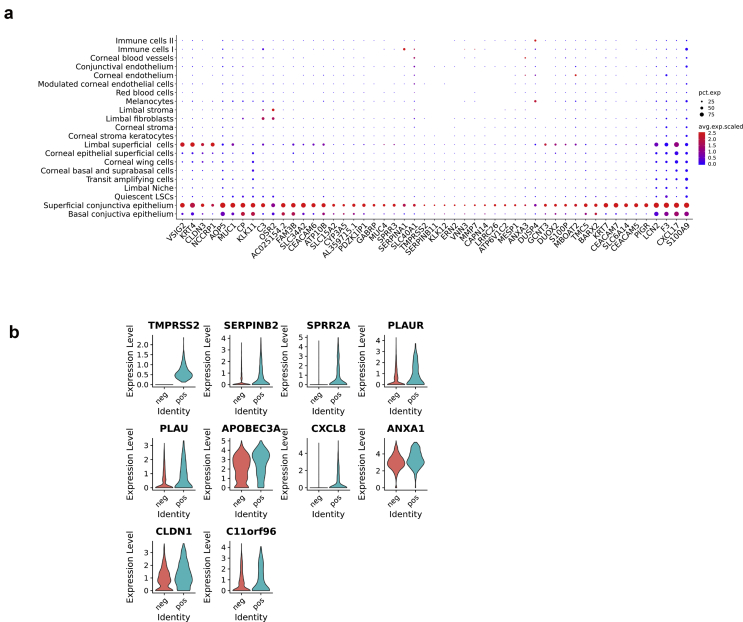
**Ocular surface genes associated with *TMPRSS2* expression and transcriptional characterisation of *TMPRSS2***^**+**^**cells**. **a)** Ocular surface epithelium expression of the top 50 genes correlated with *TMPRSS2* expression identified through Spearman's correlation analysis in the adult human corneal and conjunctiva dataset. The dot size represents the proportion of cells within the respective cell type expressing the gene and the colour represents the average gene expression level within the particular cell type; **b)** Violin expression profiles showing the expression of 10 most significantly upregulated genes in the TMPRSS2^+^ expressing cells compared to TMPRSS2^-^.

We performed similar correlation analyses for *TMPRSS2* (Extended data Fig. 4a) in the adult conjunctiva and cornea scRNA-Seq dataset. Similarly to *TMPRSS2* the top 50 *TMPRSS2* correlated genes were predominantly expressed in the superficial and basal conjunctival epithelium. These genes included *MUC1, MUC4* and *KRT4*, which are highly expressed in the superficial conjunctival epithelium alongside the complement component *C3,* which plays a central role in the activation of complement system, *CEACAM6* involved in the innate immune system [29] and *CXCL1*7 that attract immature dendritic cells and blood monocytes. Differential gene expression analysis between TMPRSS2^+^ and TMPRSS2^-^ cells (Extended data Fig. 4b) revealed interesting genes including *CXCL8* (a chemotactic factor that guides neutrophils to the site of infection), *SERPINB*2 (involved in adaptive immunity), *PLAUR*, *PLAU* and *ANXA1* (involved in innate immune response) and the DNA deaminase (cytidine deaminase) *APOBEC3A* (with restriction activity against viruses, foreign DNA and mobility of retrotransposons). Ingenuity Pathway Analysis (IPA) revealed the inflammatory responses (p = 1.03E-03 - 6.50E-11) and inflammatory diseases (p = 9.79E-04 - 1.35E-08) to be amongst the top five pathways enriched in TMPRSS2^+^ cells, which was also corroborated by the identification of lipopolysaccharide (p = 1.36E-14) and TNF (p = 3.91E-14) as upstream activating regulators. This analysis also revealed metribolone, a synthetic and orally active anabolic–androgenic steroid (AAS) and a 17α-alkylated nandrolone (19-nortestosterone) derivative and dihydrotestosterone to be the top upstream regulator (activation score 2.166) in the TMPRSS2^+^ cells in the ocular surface. AAS include male sex hormones such as testosterone and synthetic derivatives with similar structure and thus it is possible that the expression of *TMPRSS2* may be preferentially upregulated in males. Although this hypothesis remains to be validated in larger dataset, if true it may provide an additional explanation behind the increased fatality rate in men affected with COVID-19 observed in China and Italy [30].

**Fig. 4:**
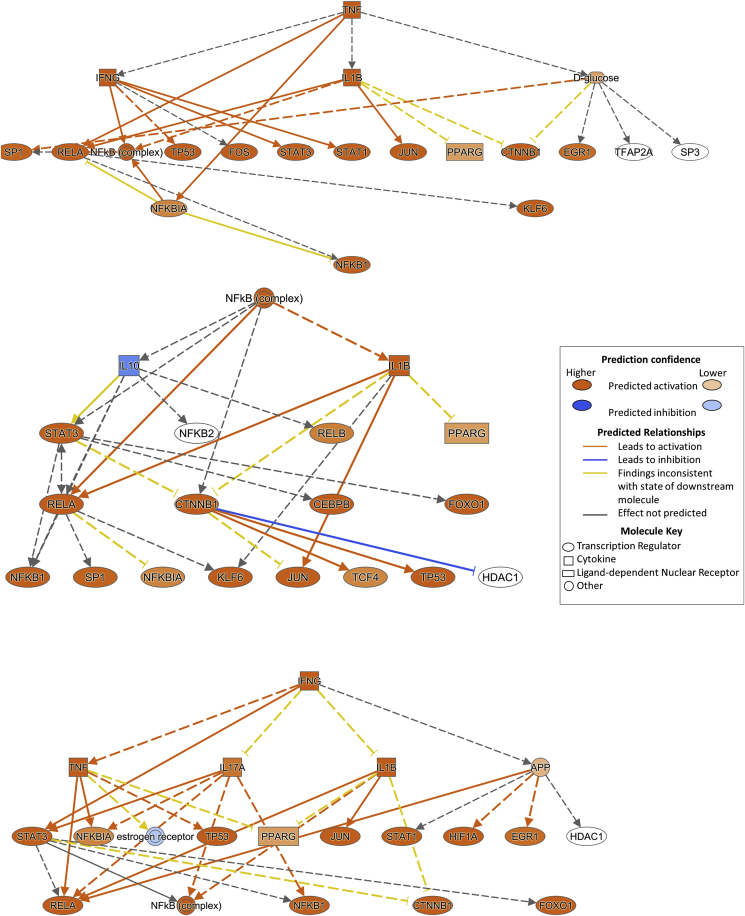
**Representative network analysis of predicted regulators in the ACE2**^**+**^**TMPRSS2**^**+**^**in the superficial conjunctival epithelial cells**. Differentially expressed genes between ACE2^+^TMPRSS2^+^ and ACE2^−^TMPRSS2^-^ cells within the superficial conjunctiva epithelium cluster were generated using the Seurat *FindMarkers* function. IPA Upstream Regulator Analysis was used to predict upstream transcriptional regulators from this gene list, using the Ingenuity® Knowledge Base to create mechanistic networks. TNF, NFkB (complex), and IFNG were predicted as upstream regulators all of which had a Z-score of greater than 3 predicting activation.

To explore the ACE2 and TMPRSS2 co-expression in a broader context, we looked further into overlaps between the 100 top associated genes for each of the two SARS-CoV-2 entry factors (Table S1). This analysis revealed the presence of 14 genes including five involved in immune response namely *FAM3D* (a regulator of neutrophil infiltration), the chemokine *CXCL17*, Cathepsin S (*CTSS*), V-Set and Immunoglobulin Domain-Containing Protein 2 (VSIG2) and Claudin 3 (*CLDN3*). Differential gene expression analysis between ACE2^+^TMPRSS2^+^ and ACE2^−^TMPRSS2^-^ cell subsets in the superficial conjunctival epithelium revealed 122 significantly changed genes (Table S2). The Regulator Effects tool of the IPA package was employed to identify potential transcriptional regulators that may explain the differential patterns of expression observed between ACE2^+^TMPRSS2^+^ and ACE2^−^TMPRSS2^-^ (Table S3). This analysis showed a large number of immune regulators predicted to activate gene expression in ACE2^+^TMPRSS2^+^ cells. The top regulators include TNF, a cytokine involved in systemic inflammation, lipopolysaccharides which are large molecules consisting of a lipid and a polysaccharide and potent stimulator of the innate immune response, NFkB (complex) a prototypical proinflammatory signalling pathway, which regulates the expression of other proinflammatory genes including cytokines, chemokines, and adhesion molecules and IL1B an important mediator of the inflammatory response and IFNG, a cytokine critical to both innate and adaptive immunity (Fig. 4). These findings were further corroborated by IPA which revealed the inflammatory response to be the top disorder (p = 3.89E-04 - 2.13E-15) predicted for ACE2^+^TMPRSS2^+^ cells in the ocular surface. In patients with COVID-19, an upregulation of pro-inflammatory cytokines such as IL-1, IL-6, TNF and IFNG has been documented [31], leading to the suggestion that controlling the cytokine storm, often observed in a sub-group of patients affected with severe COVID-19, could improve clinical outcomes and reduce the rising mortality, already observed in some small-scale trials [32]. In a very recent publication, it has been shown that *ACE2* is a human interferon stimulated gene in the airway epithelial cells [33]. It is thus very interesting to observe from the scRNA-Seq studies that these very same pro-inflammatory cytokines control the gene expression in the superficial conjunctival cells, which express both the SARS-CoV-2 *ACE2* receptor and the entry protease *TMPRSS2*. The glucocorticoid receptor signalling was identified as the top enriched pathway (p = 6.64E-05) in the ACE2^+^TMPRSS2^+^ cells. Glucocorticoids (GCs) are widely used anti-inflammatory drugs prescribed for immune and inflammatory disease such as asthma and rheumatoid arthritis. Their action is mediated by binding to the glucocorticoid receptor (GR), which upon activation translocates to the nucleus where it stimulates or inhibits gene expression. The GR mediated inhibition of pro-inflammatory pathways is achieved through the synthesis of anti-inflammatory proteins or through the repression of pro-inflammatory factors such as NF-κB or AP-1 [34]. Together these findings suggest that the ACE2^+^TMPRSS2^+^ cells are characterised by a transcriptional profile that can be activated by pro-inflammatory signalling, but at the same time capable of inhibiting the pro-inflammatory signals resulting in a feedback loop, that regulates the immune response in the ocular surface in a rather precise manner. Notwithstanding the presence of this feedback loop, the regulation of ACE2^+^TMPRSS2^+^ transcriptional profile by pro-inflammatory signals, suggests that SARS-CoV-2 may exploit inflammation-driven upregulation of ACE2 and TMPRSS2 to enhance infection in ocular surface.

### ACE2 and TMPRSS2 expression in the ocular surface during human embryonic development and childhood

**Fig. 5:**
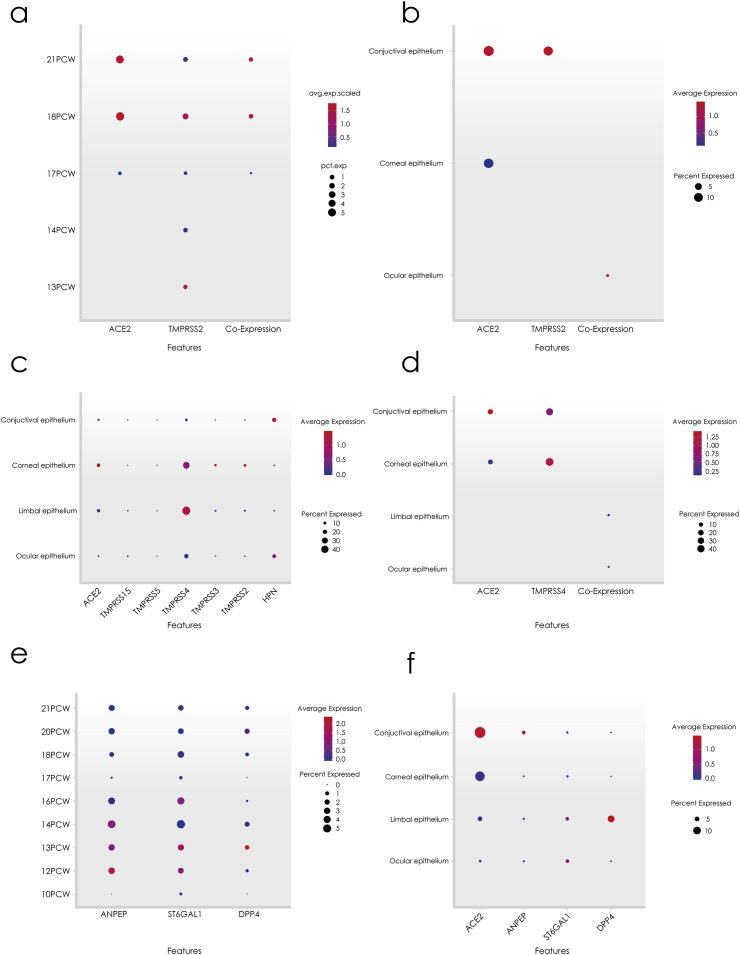
**Expression of *ACE2* and *TMPRSS2* in the embryonic and fetal human ocular surface**. **a)** RNA expression of SARS-CoV-2 receptor *ACE2* (first column), entry protease *TMPRSS2* (second column) and their co-expression (third column) during 7.2–21 PCW; **b)** RNA expression of SARS-CoV-2 receptor *ACE2* (first column), entry protease *TMPRSS2* (second column) and their co-expression (third column) in the ocular, corneal and conjunctival epithelium during human development from 7.2 to 21 PCW; **c)** RNA expression of *ACE2* and *TMPRSS* family members in the corneal, limbal and conjunctival epithelium during human development from 7.2 to 21 PCW; **d)** RNA expression of SARS-CoV-2 receptor *ACE2* (first column), protease *TMPRSS4* (second column) and their co-expression (third column) in the ocular, corneal, limbal and conjunctival epithelium during human development from 7.2 to 21 PCW; **e)** RNA expression of *ACE2*, *ANPEP*, *ST6GAL1* and *DPP4* during 7.2–21 PCW of human eye development; **f)** RNA expression of *ACE2*, *ANPEP*, *ST6GAL1* and *DPP4* in ocular, corneal, limbal and conjunctival epithelium during human development from 7.2 to 21 PCW. a-f) Raw expression values were normalised, log transformed and summarised. The size of the dots indicates the proportion of cells, while the colour indicates the mean expression.

Recent epidemiological studies present contradictory findings regarding COVID-19 infection susceptibility in children. The Chinese Centre of Disease Control and Prevention indicated 0.9% of patients to fall within the 0–9 years age group and 1.2% in the 10–19 years old with 1 overall fatality (0.2%) [35]. Similar figures were presented by Italy, with no children treated in intensive care and no deaths reported [36]. However, two recent studies have shown that children are as likely as adult to get infected with SARS-CoV-2 [37]. Despite these conflicting reports on susceptibility of children to COVID-19, all studies seem to conclude that more than 90% of affected or suspected children were asymptomatic or showed only mild to moderate symptoms. Although systematic studies need to be performed, age-related ACE2 receptor expression, lymphocyte count and trained immunity are thought to be key factors behind this increased resilience that children are showing towards SARS-Cov-2 infection [38]. To gain additional insights into *ACE2* and *TMPRSS2* expression in the ocular surface, we utilised scRNA-Seq data from embryonic and fetal corneal/conjunctival samples encompassing 7.2–21 post conception weeks (PCW). This analysis showed that *TMPRSS2* is expressed in the ocular surface from 13 PCW, while *ACE2* from 17 PCW (Fig. 5a). *ACE2* expression increased during development and was present in 4.3% of the ocular surface cells by 21 PCW, while *TMPRSS2* was much more restricted being present only in 1% of the cells.

**Extended data Fig. 5:**
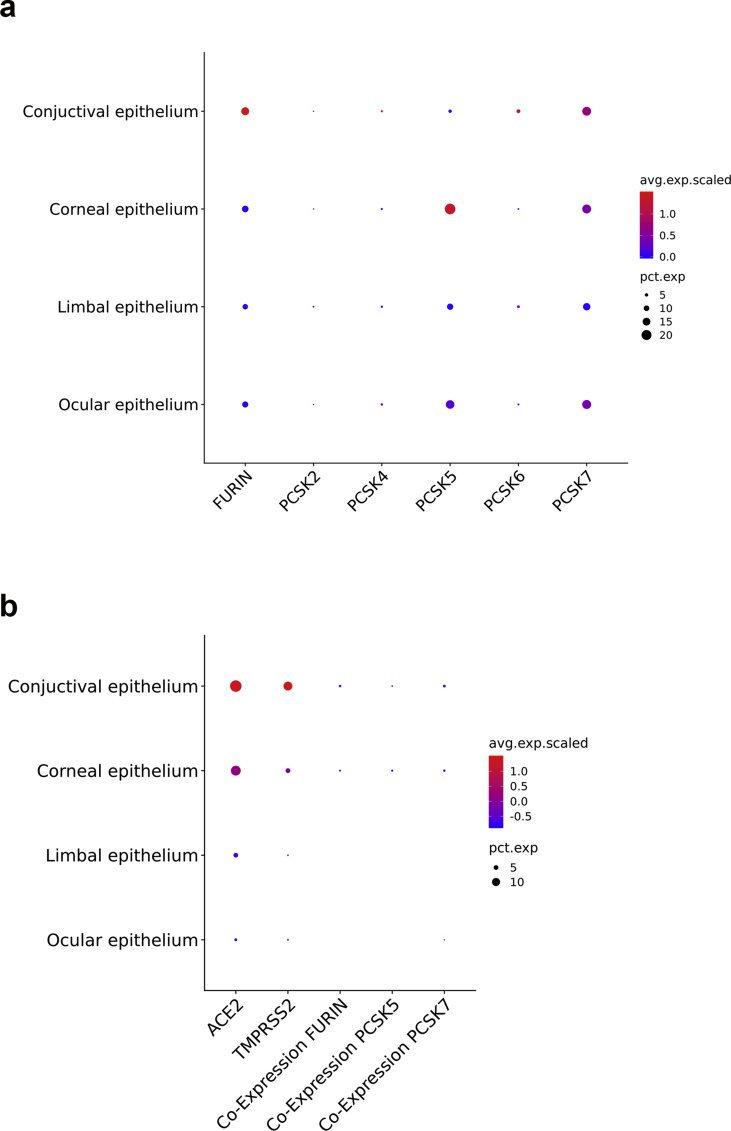
**Expression of *PCSKs* in the embryonic and fetal human ocular surface**. **a)** RNA expression of *PCSKs* during 7.2–21 PCW. **b)** RNA expression of SARS-CoV-2 receptor *ACE2* (first column), entry protease *TMPRSS2* (second column) and their co-expression with PCSKs (third column) in the ocular, corneal and conjunctival epithelium during human development from 7.2 to 21 PCW. a-b) Raw expression values were normalised, log transformed and summarised. The size of the dots indicates the proportion of cells, while the colour indicates the mean expression.

At 6.5 PCW, the developing human cornea is an extension of surface ectoderm, showing some segregation between the epithelial and stromal precursors. During 6.5–21 PCW, the ocular surface epithelium specifies into limbal, corneal and conjunctival epithelium [39]. The bioinformatic analysis of these samples showed the expression of *ACE2* in the corneal and conjunctival epithelium (Fig. 5b) and *TMPRSS2* in the conjunctival epithelium; however, co-expression of these two markers was observed in a very small subset of the remaining ocular epithelium (0.05% of the cells), which had not committed yet to limbal, corneal or conjunctival fate. *TMPRSS4* showed the highest expression in the limbal epithelium (Fig. 5c) and was co-expressed with *ACE2* in a very small subset of the remaining ocular (1.1%) and limbal (1.2%) epithelium (Fig. 5d). The expression of *ST6GAL1, ANPEP* and *DPP4* was observed from 10 PCW (Fig. 5e); nonetheless, this expression tailed off at the later developmental stages (17–21 PCW) and was much lower than *ACE2* in the conjunctival epithelium (Fig. 5f). Similarly to adult ocular surface, high expression of *CTSB* was observed in the fetal human conjuctiva cells (Extended data Fig. 2b).The expression of *PCSK3, PCSK5* and *PCSK7* was observed in the remaining ocular epithelium as well as the segregated corneal, limbal and conjunctival epithelium; however the co-expression with *ACE2* and *TMPRSS2* was observed at very low level in a very small subset of cells in the corneal and conjunctival epithelium (Extended data Fig. 5a and b).

**Extended data Fig. 6:**
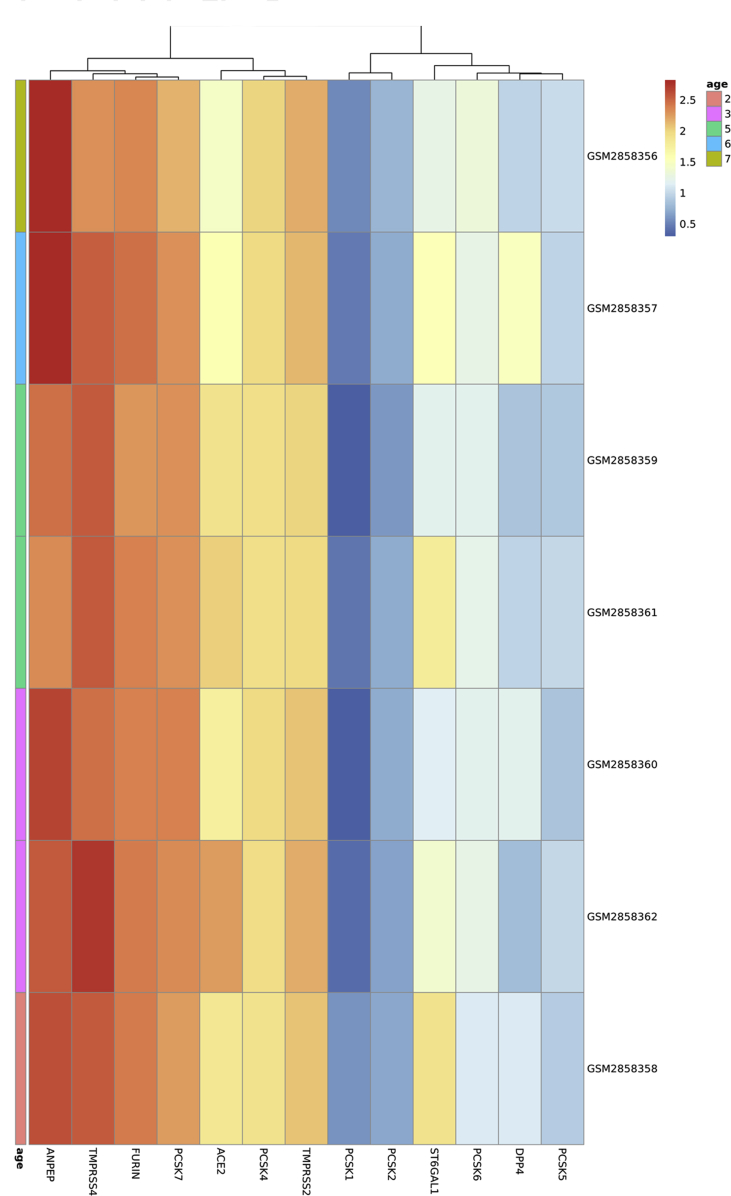
Expression heatmap analysis of the SARS-CoV-2 entry proteins in human conjunctiva of children aged to 2–7 years old.

We then investigated the expression of all potential entry genes in human bulk RNA-Seq data (GSE106961) obtained from conjunctiva of children aged between 2 and 7 years old. Moderate expression of *ACE2* and *TMPRSS2* was observed (Extended data Fig. 6), but this was lower than *TMPRSS4, PCSK3, PCSK7* and *ANPEP*. Due to nature of bulk RNA-Seq data, we were unable to compare the expression of these genes to adult or fetal conjunctiva samples.

Together our data suggest that the developing human foetuses do not co-express the SARS-CoV-2 entry genes in the conjunctival and corneal epithelium. Although expression of both of these genes is observed in the infants, it remains unclear whether these two genes are co-expressed specifically in the conjunctival or corneal epithelium.

## Conclusions

Our findings implicate the superficial ocular surface epithelium and as an additional entry route for SARS-CoV-2, which may exploit the mucus rich ocular surface to make its way into the respiratory tract via the naso-lacrimal system and/or blood vessels. Our data also suggest that SARS-CoV-2 may exploit inflammatory driven upregulation of *ACE2* and *TMPRSS2* to enhance infection in the ocular surface. The evidence presented herein has profound implications on provision of eye protective equipment for all healthcare professionals looking after patients suspected or confirmed SARS-CoV-2 positive, particularly in the ophthalmology clinics and more importantly the development of “eye drops”, which can be used by everyone as a prophylactic measure to stop viral entry through the ocular surface epithelium. This is particularly important for elderly and ‘at risk’ groups currently in isolation, but face the need for regular contact with asymptomatic carers looking after them and the risk of a potential second outburst next winter and/or when the “social distancing” and isolation measures are relaxed. While this manuscript was under preparation, a clinical case report of a Chinese COVID-19 patient who travelled from Wuhan to Italy and who presented with nonproductive cough, sore throat, coryza, and bilateral conjunctivitis was described [40]. Viral RNA was detected in ocular swabs from day 3 to day 21; moreover, SARS-CoV-2 RNA was detected again in the ocular swab at day 27, five days after it became undetectable. The viral RNA was present at lower levels than in the nasal swabs, which suggests a sustained replication in the conjunctiva. Together these findings suggest early but prolonged detection of SARS-CoV-2 in the ocular swabs and the possibility that ocular mucosa may be not only a site of virus entry but also a source of infection.

## Author contributions

JC, RQ, DZ, BD, MG, ID, RH, JMC generated the data; AJ, PR and SL provided tissue; JC, RQ, BD and ML analysed the data; JC, RQ, FCF, LA and ML interpreted the data; ML with input from JC, RQ, LA and FCF wrote the paper. All authors read the manuscript, offered feedback and approved the final version before submission.

## Declaration of competing interest

None.

## Appendix

### Materials and methods

#### Single cell sequencing and data analysis

Cornea-conjunctival tissue was excised from 4 human adult donor eyes (51, 75, 83 and 86 years old) and 13 developing human eyes from 10 to 21 post-conception weeks. The cornea-conjunctival tissues were dissociated to single cells using a multi tissue dissociation kit (Miltenyi Biotec). For scRNA-Seq cells were captured using the Chromium Single Cell 3′ Library & Gel Bead Kit, version 3 (10x Genomics). For scATAC-Seq a nuclei preparation from the dissociated cells was performed and the subsequent nuclei were captured using the Chromium Single Cell ATAC Library & Gel Bead Kit, version 1 (10x Genomics). scRNA-Seq libraries were sequenced to 50,000 reads per cell and scATAC-Seq libraries to 25,000 reads per nucleus on an Illumina NovaSeq 6000.

The sequenced samples were de-multiplexed and aligned to human reference genome GRCh38 before being quantified using CellRanger version 3.01. Quality control filtering was applied to remove any cells where fewer than 1000 reads or 500 genes or greater than 15% mitochondrial reads. 21,343 and 72,418 cells were obtained from the adult and developmental corneas/conjunctiva after data integration.

The Seurat R package (version 3.1.3) was used to normalise the individual experiments using the “LogNormalize” method. The Seurat standard integration approach was used to combine samples from developmental stage and for the adult samples. The “findVariableFeatures” used to select 2000 genes, which were used to integrate the datasets. The first 30 principle components were used for integration. The integrated datasets were then clustered using a resolution of 0.5. The *findMarkers* function, to identify markers for each cluster. Cell types were assigned to these clusters were annotated using these genes lists. Cluster identity was validated using immunohistochemistry (unpublished data). The clustering results were visualised using uniform manifold approximation and projection (UMAP). Scaled expression values below zero, which equated to 0–1 counts were filtered from co-expression analysis.

Differential expression analysis was used to compare ACE2+TMPRSS2+ cells within “superficial conjunctiva” to the rest of the cells within this cluster. The gene lists were analysed using the upstream regulator function from QIAGEN Ingenuity Pathway Analysis (QIAGEN IPA).

Spearman's Rank Correlation analysis was used to find the top 50 genes with highest correlation of expression to *ACE2* and *TMPRSS2*.

CellPhoneDB was used to predict interacting cells across clusters. The analysis was performed using the clusters identified by Seurat. Additionally the “superficial conjunctiva” was split into ACE2+TMPRSS2+ versus negative populations to look for interacting cell types.

CellRanger ATAC (version 1.2) used to identify accessible regions from scATAC-Seq sequencing files. The cellranger-atac agrr module was used to create a set of shared peaks present across all four samples. The samples were then analysed with Signac version 0.2.4 along with Seurat. The percentage of reads in peaks, number of reads in percentage of reads in peaks per cell, percentage mapping to ENCODE blacklist, nucleosome signal and TSS enrichment was calculated. The data was filtered using thresholds to remove cells where the number of fragments within peaks was less than 1000 or greater than 50000, or with a blacklist ration of greater than 5% or a nucleosome signal of greater 10 and a TTS enrichment of greater than 2. A gene activity matrix was calculated in the ATAC-Seq dataset, using the closest genes to the peaks. Seurat was then used to perform clustering analysis on each sample and transfer the cell type labels from RNA-Seq to ATAC-Seq data. Seurat integration methods were applied to the data peak accessibility to overcome donor effects and combine the four ATAC-Seq datasets. Signac was used to create “pseudo-bulk” accessibility tracks by grouping cells by cell type within promoter/enhancer regions of key genes.

Seven infant conjunctival samples ("GSM2858356", "GSM2858357", "GSM2858358", "GSM2858359", "GSM2858360", "GSM2858361", "GSM2858362") were downloaded from GSE106961 using the GEOquery R package. The donors ranged from 2 to 7 years old. The normalised log2 expression values of *ACE2, TMPRSS2, TMPRSS4, PCSK3, PCSK7* and *ANPEP* were compared in these samples.

#### Immunohistochemistry

Adult cornea/conjuctival sections were incubated in 4% PFA overnight, followed by three washes in Phosphate-buffered saline (PBS), incubated overnight in 30% sucrose in PBS, embedded in Optical Cutting medium (OCT) (Cellpath, UK) and frozen at −20 °C. Ten-micrometre cryostat sections were collected using a Leica Cm1860 cryostat (Leica, Germany) onto Superfrost Plus slides and stored at −20 °C in slide boxes prior to immunostaining. Cryosections were air-dried, washed several times in PBS and incubated in blocking solution (10% normal goat serum, 0.3% Triton-X-100 in PBS) for 1 h at room temperature. Slides were incubated with ACE2 (Abcam, ab15348, 1:200 dilution), ACE2 (AF933, R&D Systems, 1: 200 dilution), TMPRSS2 (ab92323, Abcam 1:500), MUC4 (Abcam, ab60720, 1:200 dilution) and MUC5AC (ab24070, Abcam, 1: 200 dilution) overnight at 4 °C. After rinsing with PBS, sections were incubated with the secondary antibodies for 2 h at room temperature (goat anti mouse alexa488, cat number 115-545-062; goat anti rabbit Cy3, cat-number 111-165-144, both from Jackson Immunoresearch and used at 1:1000 dilution). Negative controls were carried out by omitting the primary antibody. Afterwards, sections were washed three times in PBS and mounted with Vectashield (Vector Laboratories, Burlingame, CA) containing Hoechst (Life Technologies, UK). Images were captured using an Axio Imager upright microscope with Apotome (Zeiss, Germany) structured illumination fluorescence using 20x objective and 63x oil objective operated with AxioVision software. Final images are presented as a maximum projection and adjusted for brightness and contrast in Adobe Photoshop (Adobe Systems).

#### Western Blotting

Cornea or conjunctival tissue was homogenised in RIPA buffer with protease inhibitors (cOmplete Protease Inhibitor Cocktail, Roche). Insoluble material was removed by centrifugation at 10,000 g for 15 min at 4 °C. Protein quantification was performed on the lysate (BCA Assay, Pierce). 10 μg of protein extract was added to lithium dodecyl sulphate sample loading buffer with sample reducing agent (NuPAGE, ThermoFisher Scientific) and boiled for 5 min at 95 °C, followed by cooling on ice for 10 min. Reduction of the protein samples by polyacrylamide gel electrophoresis (NuPAGE 4–12% Bis-Tris 1.0-mm minigel, NP0322, ThermoFisher Scientific) alongside a protein marker (PageRuler Plus prestained protein ladder, 26619, ThermoFisher Scientific) was performed. Resolved proteins were then transferred onto a polyvinylidene difluoride membrane using an iBlot2 system (IB24002, ThermoFisher Scientific). The membrane was blocked with 5% non-fat milk/tris-buffered saline, followed by overnight incubation with either anti-ACE2 (1 μg/mL, AF933, R&D Systems), anti-TMPRSS2 (1:1000, ab92323, Abcam) or anti-GAPDH (1:1000, sc-47724, Santa Cruz Biotechnology) primary antibody at 4 °C, washing for 5 min at least 3 times in 0.1% Tween-20/tris-buffered saline, and incubating with the appropriate HRP-conjugated secondary antibodies; either swine anti-Rabbit (1:2000, P0399, Agilent Dako) or anti-Mouse (1:2000, P0447, Agilent Dako) for 1 h at room temperature. Immunoreactivity was visualised with SuperSignal West Pico PLUS (ThermoFisher Scientific) chemiluminescence reagent.

Data availability statement: All the datasets were generated in Lako's lab are currently unpublished: these can be assessed and visualised through the website portal (http://covid19ocularsurface.org/).

## Appendix A Supplementary data

Table S1 **Immune genes associated with *ACE2* and *TMPRSS2* expression**. Immune associated genes in human adult ocular surface associate from the top 100 genes correlated with *ACE2* and *TMPRSS2*. Table S1

Table S2 Differential gene expression between ACE2^+^TMPRSS2^+^ versus ACE2^−^TMPRSS2^-^ cells. List of significantly and differentially expressed genes between ACE2^+^TMPRSS2^+^ versus ACE2^−^TMPRSS2^-^ cells. Table S2

Table S3 List of significant regulators of gene expression in the ACE2^+^TMPRSS2^+^ cells in the superficial conjunctival epithelium. Table S3
