# Supplementary material for: Co-expression of SARS-CoV-2 entry genes in the superficial adult human conjunctival, limbal and corneal epithelium suggests an additional route of entry via the ocular surface
Source: Ocul Surf. 2021 Jan;19:190–200. doi: 10.1016/j.jtos.2020.05.013 (PMC7267807; doi:10.1016/j.jtos.2020.05.013)
Supplement: Table S1 — Immune genes associated with ACE2 and TMPRSS2 expression. Immune associated genes in human adult ocular surface associate from the top 100 genes correlated with ACE2 and TMPRSS2. [file mmc1.docx]

| **Genes** | **Name** | **Role** | **Relevant Pathcards** |
| --- | --- | --- | --- |
| FAM3D | FAM3 Metabolism Regulating Signaling Molecule D | Regulated neutrophil infiltration in the aorta. |  |
| PSCA | Prostate Stem Cell Antigen | Glycoprotein required at later stages of corneal development and involved in maintenance of the epithelial layer. | [Post-translational modification- synthesis of GPI-anchored proteins](https://pathcards.genecards.org/Card/post-translational_modification-_synthesis_of_gpi-anchored_proteins?queryString=msln)  Metabolism of proteins |
| BCAS1 | Breast Carcinoma Amplified Sequence 1 |  |  |
| MSLN | Mesothelin | Glycosylphosphatidylinositol-anchored cell-surface protein that may function as a cell adhesion protein. | [Post-translational modification- synthesis of GPI-anchored proteins](https://pathcards.genecards.org/Card/post-translational_modification-_synthesis_of_gpi-anchored_proteins?queryString=msln)  Adhesion  Metabolism of proteins |
| HOPX | HOP Homeobox | Atypical homeodomain protein, which does not bind DNA and expressed in stratified epithelia. |  |
| NCCRP1 | NCCRP1, F-Box Associated Domain Containing | Promotes cell proliferation. |  |
| CXCL17 | C-X-C Motif Chemokine Ligand 17 | A mucosal chemokine that attracts immature dendritic cells and blood monocytes to the lungs. May play a role in the innate defence against infections. Activates the C-X-C chemokine receptor GPR35 to induce a rapid and transient rise in the level of intracellular calcium ions. | C[hemokine Superfamily Pathway: Human/Mouse Ligand-Receptor Interactions](https://pathcards.genecards.org/Card/chemokine_superfamily_pathway_humanmouse_ligand-receptor_interactions?queryString=CXCL17)  [PEDF Induced Signaling](https://pathcards.genecards.org/Card/pedf_induced_signaling?queryString=CXCL17) |
| SMIM22 | Small Integral Membrane Protein 22 | May modulate lipid droplet formation through interaction with SQLE. |  |
| PRSS22 | Serine Protease 22 | A member of the trypsin family of serine proteases. The enzyme is expressed in the airways in a developmentally regulated manner. |  |
| VSIG2 | V-Set And Immunoglobulin Domain Containing 2 | An important paralog of this gene is [ESAM](https://www.genecards.org/cgi-bin/carddisp.pl?gene=ESAM), (Endothelial Cell Adhesion Molecule) is a Protein Coding gene. | T cell activation, including IL17 and interferon gamma production |
| RAB11FIP1 | RAB11 Family Interacting Protein 1 | Plays a role in the Rab-11 mediated recycling of vesicles. May be involved in endocytic sorting, trafficking of proteins and transport between the recycling endosome and the trans-Golgi network. | [Endocytosis](https://pathcards.genecards.org/Card/endocytosis?queryString=RAB11FIP1)  [Cytoskeletal Signaling](https://pathcards.genecards.org/Card/cytoskeletal_signaling?queryString=RAB11FIP1) |
| CTSS | Cathepsin S | A lysosomal cysteine proteinase that may participate in the degradation of antigenic proteins to peptides for presentation on MHC class II molecules. The protein can function as an elastase over a broad pH range in alveolar macrophages. | [Lysosome](https://pathcards.genecards.org/Card/lysosome?queryString=ctss)s  [Innate Immune System](https://pathcards.genecards.org/Card/innate_immune_system?queryString=ctss)  Activated TLF4 signaling  [MHC class II antigen presentation](https://pathcards.genecards.org/Card/mhc_class_ii_antigen_presentation?queryString=ctss)  [Phagosome](https://pathcards.genecards.org/Card/phagosome?queryString=ctss)  [Class I MHC mediated antigen processing and presentation](https://pathcards.genecards.org/Card/class_i_mhc_mediated_antigen_processing_and_presentation?queryString=ctss) |
| CLDN3 | Claudin 3 | An integral membrane protein and a component of tight junction strands expressed in the corneal epithelium. Plays a major role in tight junction-specific obliteration of the intercellular space, through calcium-independent cell-adhesion activity. It is also a low-affinity receptor for Clostridium perfringens enterotoxin. | [Cell adhesion_Endothelial cell contacts by junctional mechanisms](https://pathcards.genecards.org/Card/cell_adhesion_endothelial_cell_contacts_by_junctional_mechanisms?queryString=cldn3)  [Pathogenic Escherichia coli infection](https://pathcards.genecards.org/Card/pathogenic_escherichia_coli_infection?queryString=cldn3)  [Toll-like Receptor Signaling Pathway](https://pathcards.genecards.org/Card/toll-like_receptor_signaling_pathway?queryString=cldn3)  [Blood-Brain Barrier and Immune Cell Transmigration: VCAM-1/CD106 Signaling Pathways](https://pathcards.genecards.org/Card/blood-brain_barrier_and_immune_cell_transmigration_vcam-1cd106_signaling_pathways?queryString=cldn3)  [Cytoskeletal Signaling](https://pathcards.genecards.org/Card/cytoskeletal_signaling?queryString=cldn3) |
| TTC9 | Tetratricopeptide Repeat Domain 9 | Has been shown to be hormonally regulated in breast cancer cells and may play a role in cancer cell invasion and metastasis. |  |
